# Invasive micropapillary breast carcinoma: A retrospective study on the clinical imaging features and pathologic findings

**DOI:** 10.3389/fsurg.2022.1011773

**Published:** 2022-09-23

**Authors:** Jiarui Nangong, Zhongquan Cheng, Leyi Yu, Xiaodan Zheng, Guoqian Ding

**Affiliations:** ^1^ Department of General Surgery, Beijing Friendship Hospital, Capital Medical University, Beijing, China; ^2^ Departament of Chronic Wound Repair Surgery, Beijing Haidian Hospital, Peking University, Beijing, China; ^3^ Department of Pathology, Beijing Friendship Hospital, Capital Medical University, Beijing, China

**Keywords:** invasive micropapillary breast carcinoma, mammography, sonography, MRI, molecular imaging

## Abstract

**Purpose:**

To describe the clinical imaging and pathological features of invasive micropapillary breast carcinoma (IMPC), including breast mammography, sonography, magnetic resonance imaging (MRI), and molecular imaging findings.

**Patients and methods:**

We retrospectively reviewed our institution's surgical pathology database and identified 65 patients with pathologically proven IMBC; 63/65 patients had available imaging results. Two radiologists retrospectively reviewed all imaging evaluations according to the Breast Imaging Reporting / Data System (BI-RADS) Lexicon. Clinical and histopathologic features, receptor statuses, and clinical follow-up data were recorded.

**Results:**

Sixty-three patients were admitted with palpable abnormalities; one patient's mammogram revealed no abnormality (3.3%, 1/32), whereas 31 had abnormal mammograms (31/32, 96.8%) demonstrating 37 lesions. Twenty-four had irregular, spiculated masses, 12 had microcalcifications, and 19 had architectural distortion. Sonography detected 69 masses (54 patients), characterized by irregular shapes (61/69, 88.4%), hypoechoic structures (50/69, 72.4%), angular or spiculated margins (38/69, 55.1%; 30/69, 43.4%), echogenic halo (8/69, 11.5%), and abnormal vascularity (52/69, 75.3%). MRI detected 68 lesions (52 patients); 59/68 (86.8%) appeared as masses with angular or spiculated margins (32/68, 47.1%; 35/68, 51.4%), 58 exhibited irregular or lobulated shapes (58/68, 89.7%), 29 displayed heterogeneous internal enhancement (29/68, 42.5%), and 64 demonstrated type II or III washout kinetic curves (37/68, 55%; 27/68, 40%). Breast molecular imaging showed mild-to-moderate radiotracer uptake in 15 focal areas among 13 patients. Thirty-two, 38, and 43 patients had abnormal lymph nodes identified mammographically, by breast sonography, and by MRI, respectively. Immunohistochemistry revealed high estrogen receptor (90.5%), high progesterone receptor (71.6%), and low HER-2 (26.4%) positivity.

**Conclusion:**

IMPC mammography, sonography, and MRI clinical imaging features highly suggest malignancy. Breast molecular imaging also contributed to the diagnosis. IMPC's invasiveness correlated well with regional lymph node metastasis. Radiologists and surgeons should be more attentive to these imaging findings and additional clinical and pathological IMPC features.

## Introduction

Invasive micropapillary breast carcinoma (IMPC) is a relatively rare subtype of invasive ductal breast carcinoma, accounting for less than 5% of all breast cancer cases (1). In 1980, Fisher et al. were the first to describe the structure of IMPC as having an “exfoliative appearance” (2). Thirteen years later, IMPC was defined and included in the World Health Organization's (WHO) classification of breast tumors (3). Pathologists have described IMPC lesions as having an inside-out reversed polarity pattern with a typical cuticle-like microvillous secretory surface facing the stroma, resulting in a gap between the stroma and the neoplastic epithelial cells (4). Prior studies have demonstrated that as an aggressive variant, IMPC is linked to a larger tumor size, more advanced Tumor, Node, Metastasis (TNM) staging, a greater proportion of nodal involvement, and a higher degree of lymphovascular invasion (LVI); these characteristics can contribute to a high early recurrence rate and a poorer prognosis (5).

To date, most studies have focused on the pathologic characteristics of IMPC, and only a few studies have investigated the correlation between imaging results and the clinical and pathologic features of this disease (6–8). Understanding the radiologic features of IMPC would be helpful in distinguishing this subtype of breast cancer from others. In addition, the imaging characteristics of IMPC have been mostly reported based on mammography and sonography, whereas a limited number of studies have focused on additional MRI and molecular imaging features of IMPC (6, 7). Simultaneously, there has been a lack of comprehensive reports on the clinical imaging characteristics of IMPC over the past three years. Therefore, we aimed to retrospectively evaluate the clinical imaging findings, histopathologic results, and prognosis of patients with IMPC who underwent mammography, sonography, MRI, and molecular imaging of the breast at our institution according to the Breast Imaging Reporting / Data System (BI-RADS) Lexicon.

## Materials and methods

After receiving institutional review board approval, we reviewed patient information in our institution's surgical pathology database from January 1, 2012 through September 30, 2021 to identify cases with pathologically diagnosed IMPC. Imaging characteristics, including mammography, sonography, MRI, and molecular imaging of the breast, were evaluated by two radiologists specializing in breast imaging. The clinical presentation was also reviewed in each case and correlated with imaging and pathologic information. A breast pathologist reviewed and confirmed the diagnostic features of all cases included in this study.

### Patient population

We surveyed 4,162 patients with pathologic reports confirming breast cancer diagnoses between January 2012 and September 2021; ultimately, we successfully identified 65 patients with variable proportions of IMPC components. Due to a lack of available imaging information for two patients, a total of 63 cases were finally included in our study.

### Breast mammography

Two standard mammographic views with craniocaudal and mediolateral oblique projections were obtained for all patients, and additional views were collected when the radiologists deemed it necessary based on a mammographic abnormality. Dedicated screen-film mammographic equipment was used (Senograph DS VERSION ADS-53.40, GE Healthcare). The lesion-based evaluation of the abnormalities included the following characteristics: the shape, margin, and size of the mass; the distribution and morphologic features of microcalcifications; and architectural distortion with associated nipple retraction, skin thickening, and axillary adenopathy.

### Breast sonography

Breast sonography was performed with a Philips IU 22 breast ultrasound system (Philips Healthcare, Eindhoven, Netherlands) using a high-resolution ultrasound scanner and a high frequency (10–14 MHz) linear array transducer. The shape, margin, size, posterior acoustic features, and echogenicity of each lesion were all evaluated according to the American College of Radiology (ACR) BI-RADS final assessment. The largest diameter obtained from either the sagittal or transverse view was reported as the maximum diameter of each breast tumor. Axillary abnormalities were also reviewed, including eccentric cortical thickening or lymph node replacement.

### Breast MRI

Breast MRI examinations were performed using a commercially available SIGNA Pioneer 3.0-T whole-body imaging system (GE Healthcare Life Sciences, Buckinghamshire, England) with a dedicated breast coil. All patients were scanned in the prone position, with images acquired in both the sagittal and axial planes. Contrast-enhanced T1-weighted 3D fat-suppressed spoiled gradient-echo images (TR/TE, 4.7/2.3; flip angle, 10°; bandwidth, 62.5 kHz) were obtained 90 s before and three times after rapid intravenous injection of 0.1 mmol/L of gadobenate dimeglumine (Multihance, Bracco Diagnostics) per kilogram of body weight at a rate of 2 ml/s. Image acquisition was initiated after contrast administration. Lesion-based evaluations included the characteristics of the mass, the associated kinetics, and non-mass enhancement. Associated findings were also reported, including, but not limited to, skin thickening, chest wall invasion, and axillary adenopathy.

### Molecular breast imaging

Molecular breast imaging was performed in 13 patients after at least 8 h of fasting. Each patient received an intravenous injection containing 370–450 MBq of ^18^F-fluorodeoxyglucose (FDG) and were hydrated with 500 ml of intravenous saline (0.9% sodium chloride). One hour after ^18^F-FDG administration, positron emission tomography/CT (PET/CT) was performed for each breast over a 10-minute period in the craniocaudal and mediolateral oblique projections using a PET/CT Discovery IQ system (GE Medical Systems, Fairfield, CT, USA).

### Image analysis

Two trained radiologists with eight and ten years of experience in breast imaging, respectively, reviewed all clinical images from patients with IMPC. According to the ACR BI-RADS criteria, the mammographic, sonographic, and MRI characteristics were evaluated in all cases. The molecular breast imaging findings of IMPC cases were retrospectively reviewed based on a validated molecular breast imaging lexicon (9).

### Surgical procedure

The patients with IMPC lesions underwent various surgical procedures for treatment. The surgical modalities were chosen according to each patient's risk and preference, clinical tumor staging, and lymph node status.

### athologic information

Data on all of the IMPC lesions were available and included in our study. A breast pathologist evaluated the tumor type and size, the Nottingham histological grade, the absence or presence of LVI, the estrogen receptor (ER), progesterone receptor (PR), and human epidermal growth factor receptor 2 (HER2) receptor statuses, and the axillary lymph node status.

## Results

### Patient characteristics

Our study successfully identified 65 patients whose ages ranged from 28 to 90 years (mean, 56.3 years), with a micropapillary component ratio between 10% and 100%. The final analysis included 63 patients with available clinical and imaging data, all of whom were women. Of the 63 patients, 65 lesions were diagnosed; two had bilateral diseases, whereas the rest of the cases were unilateral (29 lesions on the left and 32 on the right). All patients underwent standardized physical examinations. Palpable abnormalities were identified in 47 patients (47/63, 74.6%), including 30 with a palpable mass (30/63, 48%), 12 with nipple retraction (12/63, 19%), 14 with skin abnormalities (14/63, 22.2%), five with breast pain (5/63, 8%), and 16 (16/63, 25%) without obvious discomfort upon examination. The main characteristics of these patients are summarized in Table 1.

**Table 1:** Patient characteristics (*n* = 63).

Characteristics	Values
**Sex**	
Male	0 (0)
Female	63 (100)
Age (year), mean (range)	56.3 (28–90)
No obvious clinical discomfort	16 (25)
**Clinical presentation**	
Palpable mass	30 (48)
Lesion location	
Left side	29 (46)
Right side	32 (51)
Both sides	2 (3)
Screening performed	
Mammography	32 (51)
Sonography	54 (86)
Magnetic resonance imaging (MRI)	52 (83)
Molecular imaging	13 (21)
Nipple retraction	12 (19)
Skin abnormalities	14 (22)
Breast pain	5 (8)

Notes: Except for age, data are represented as no. (%) of patients.

### Breast mammography characteristics

**Figure 1:**
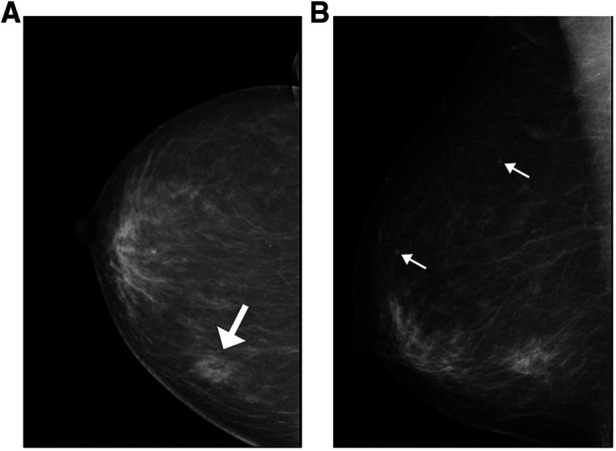
Mammographic findings of patients with invasive ductal carcinoma with micropapillary features (**A**) a 74-year-old woman with a palpable abnormality in the left breast. A mass is visible in the posterior portion of the central area of the left breast, measuring approximately 1.7 cm × 1.5 cm × 1.8 cm; it is lobulated, with burrs visible at the edges and tiny calcifications visible within it (white arrow indicates the location of the breast mass). (**B**) A 74-year-old woman with a palpable abnormality in the right breast and flocculent high-density shadowing in the lower quadrant. Associated pleomorphic microcalcifications are also visible within the breast (white arrows indicate the locations of microcalcification).

Among the 63 patients included in the analysis, 32 underwent breast mammography; the mammographic findings are summarized in Table 2. A total of 37 lesions were identified in 32 patients. The breast mammogram showed no abnormality in one patient (1/32, 3.3%); the mass was impalpable and was identified *via* breast sonography and MRI. The breast mammograms detected more than one abnormality in three patients (one patient with two lesions and two with three lesions). The average length of all masses detected mammographically was 2.3 cm (range, 0.9 cm–6.5 cm). Masses were mostly detected as an irregular shape (24/37, 64.8%) with spiculation (19/37, 51.3%) or indistinct margins (11/37, 29.7%) and high density (22/37, 59.4%) (Figure 1A). Twelve masses presented with associated microcalcifications (Figure 1B); 10 of these exhibited a punctate morphology (10/12, 83.3%), with nine lesions demonstrating a scattered distribution (9/12, 75%). One lesion presented with segmental asymmetry (1/12, 8.3%).

**Table 2:** Mammographic characteristics of IMPC lesions (*n* = 32).

Characteristics	No. (%)
Negative	1 (3.3)
(Focal) asymmetry	6 (18.7)
Calcification only	0 (0)
Mass	
Shape (*n* = 32)	
Round	2 (6.3)
Oval	5 (15.6)
Lobular	4 (12.5)
Irregular	21 (65.6)
Margin (*n* = 32)	
Circumscribed	3 (9.4)
Microlobulated	3 (9.4)
Obscured	1 (3.1)
Indistinct	9 (28.1)
Spiculated	16 (50)
Density (*n* = 32)	
High	19 (59.4)
Equal	1 (3.1)
Low	0 (0)
Mixed	12 (37.5)
Calcifications	
Shape (*n* = 12)	
Amorphous	2 (16.7)
Round	0 (0)
Punctate	10 (83.3)
Fine Pleomorphic	0 (0)
Fine linear or fine linear branching	0 (0)
Distribution (*n* = 12)	
Regional	2 (16.7)
Clustered	0 (0)
Linear	0 (0)
Segmental	1 (8.3)
Scattered	9 (75)

Notes: Data represent the no. (%) of patients.

Abbreviations: IMPC, invasive micropapillary breast carcinoma.

### Breast sonography characteristics

**Figure 2:**
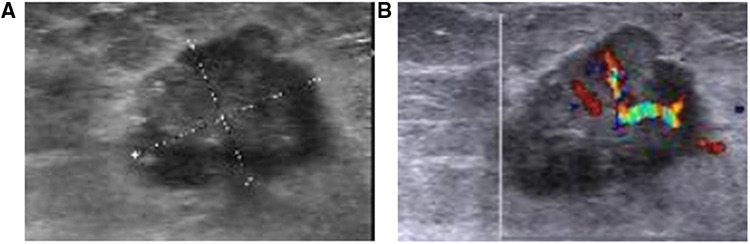
Breast sonography from a 42-year-old woman with a suspicious breast mass (**A**) the breast sonography shows a hypoechoic mass in the left breast, located 1 cm away from the nipple in the 6 O'clock direction, with a spiculated margin, irregular shape, and crab foot sign. (**B**) Color Doppler sonography shows the presence of large blood vessels in the tumor, with obvious arterial blood flow signals.

Breast sonography was performed in 54 patients and successfully detected a total of 69 lesions, the sonographic features of which are presented in Table 3. Ten patients had more than one lesion (six patients had two, three had three, and one had four, respectively). The mean tumor size detected sonographically was 2.1 cm (range, 0.8–5.0 cm). Among the 69 lesions, 50 masses were identified with hypoechoic (50/69, 72.4%) characteristics, and 16 had complex posterior acoustic characteristics (16/69, 23.1%). Breast sonography also revealed that the masses predominantly displayed an irregular shape (61/69, 88.4%), with spiculated or angular margins (38/69, 55.1%; 30/69, 43.4%), and an echogenic halo (8/69, 11.5%) (Figure 2A). Color Doppler interrogation was performed in 52 out of 69 lesions; 57.7% (30/52) of the lesions exhibited punctate blood flow, whereas 23.1% (12/52) showed striped blood flow patterns (Figure 2B). Both breast mammography and sonography were performed in 29 patients. The breast sonography identified 37 lesions, whereas only 31 were detected *via* mammography (16.2% more lesions detected by breast sonography).

**Table 3:** Sonographic characteristics of IMPC lesions (*n* = 54).

Characteristics	No. (%)
Shape	
Oval	4 (7.4)
Round	2 (3.7)
Irregular	48 (88.9)
Orientation	
Parallel	22 (40.7)
Not parallel	32 (59.2)
Margin	
Circumscribed	1 (1.9)
Indistinct	0 (0)
Angular	23 (42.5)
Microlobulated	0 (0)
Spiculated	30 (55.6)
Lesion boundary	
Abrupt interface	13 (24.1)
Echogenic halo	41 (75.9)
Echo pattern	
Hyperechoic	0 (0)
Isoechoic	2 (3.7)
Hypoechoic	39 (72.2)
Complex	12 (22.2)
Anechoic	1 (1.9)
Posterior acoustic features	
No posterior acoustic feature	2 (3.7)
Enhancement	20 (37.0)
Shadowing	11 (20.3)
Combined pattern	21 (38.9)
Color Doppler	Done in 52/69 lesions
0	10 (19.2)
+	30 (57.7)
++	12 (23.1)

Notes: Data represent the no. (%) of patients.

Abbreviations: IMPC, invasive micropapillary breast carcinoma.

### Breast MRI characteristics

**Figure 3:**
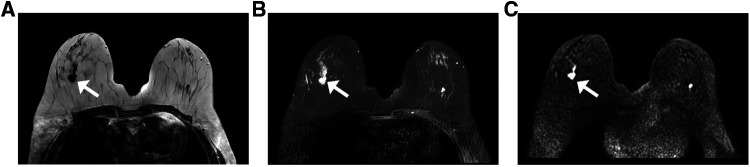
MRI from a 63-year-old woman with a palpable abnormality in the right breast the MRI shows an irregular mass with a spiculated margin in the upper outer quadrant. The tumor exhibits the following characteristics: (**A**) a low signal on the T1WI; (**B**) a high signal on the fat-suppressed T2WI; and (**C**) a high signal on the DWI (white arrows indicate the locations of lesions). Abbreviations: MRI, magnetic resonance imaging; T1WI, T1-weighted imaging; T2WI, T2-weighted imaging; DWI, diffusion-weighted imaging.

Breast MRI can be used for staging assessment to determine the extent of ipsilateral breast tumor and the presence of a multifocal or multicentric tumor. Furthermore, it helps to assess tumor extent before and after neoadjuvant therapy, the response to therapy, and the availability of breast-conserving therapy. The examinations were performed for cancer staging in 52 patients and identified a total of 68 lesions (eight patients had two lesions, four had three lesions, all other only have one lesion). The mean tumor size detected *via* MRI was 2.0 cm (range, 0.8 cm–5.9 cm). Twenty-seven patients underwent breast mammography, sonography, and MRI examinations, which revealed 31, 34, and 35 lesions, respectively. The MRI findings of the lesions are summarized in Table 4. Fifty-three lesions appeared as irregularly shaped masses (53/68, 77.9%), with irregular or spiculated margins (23/68, 33.8%; 30/68, 44.1%), displaying homogenous enhancement (17/68, 25%), heterogeneous enhancement (29/68, 42.5%), and rim enhancement (22/68, 32.5%), with type II or III kinetic curves (37/68, 55%; 27/68, 40%) (Figure 3). Non-mass-like enhancement was identified in seven patients with segmental distribution, six of whom (85.7%) exhibited heterogeneous internal enhancement. Associated findings, including nipple retraction (17.3%, 9/52) and diffuse skin thickening (26.9%, 14/52), were also evident in breast MRI.

**Table 4:** MRI characteristics of IMPC lesions (*n* = 52).

Characteristics	No. (%)
Mass	47
Shape	
Oval	2 (4.3)
Lobular	4 (8.5)
Irregular	41 (87.2)
Margin	
Smooth	1 (2.1)
Irregular	22 (46.8)
Spiculated	24 (51.1)
Non-mass	5 (9.6)
Pattern	
Segmental	5 (100)
Multiple regions	0 (0)
Internal enhancement	
Homogenous	1 (20.0)
Heterogenous	4 (80.0)
Kinetic curve assessment at delayed phase	
Persistent	3 (5.8)
Plateau	29 (55.8)
Washout	20 (38.5)

Notes: Data represent the no. (%) of patients.

Abbreviations: MRI, magnetic resonance imaging; IMPC, invasive micropapillary breast carcinoma.

### Breast molecular imaging findings

**Figure 4:**
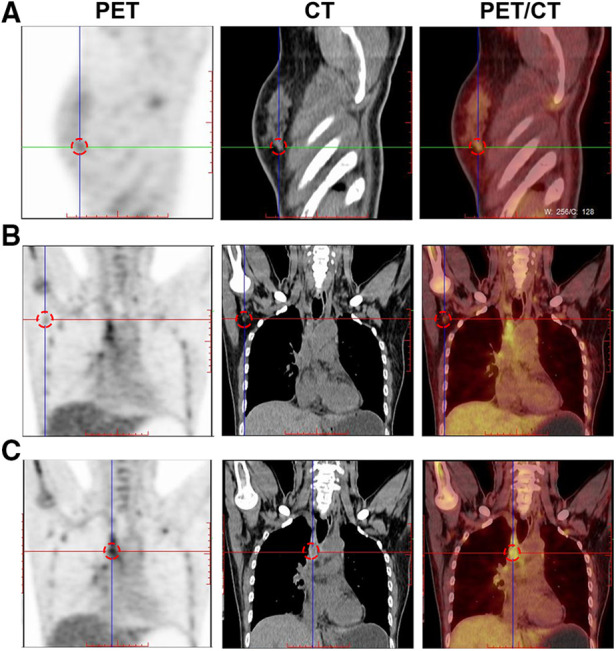
Breast molecular imaging findings from patients with invasive ductal carcinoma with micropapillary features (**A**) A soft, irregular tissue nodule is seen in the lower outer quadrant of the right breast, approximately 1.0 cm × 0.8 cm in size, exhibiting abnormal increased uptake of ^18^F-FDG. (**B**) Several small lymph nodes are visible in the right axilla. The larger ones are approximately 1.2 cm × 0.7 cm in size and exhibit mildly increased uptake of ^18^F-FDG. Abbreviations: PET, positron emission tomography; CT, computed tomography; ^18^F-FDG, ^18^F-fluorodeoxyglucose radiotracer.

**Figure 5:**
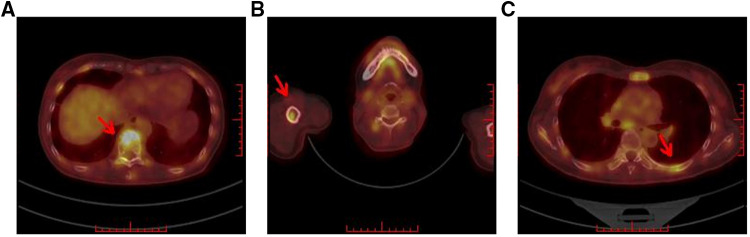
Breast molecular imaging findings from a 50-year-old woman with invasive ductal carcinoma with micropapillary features diagnosed with distant bone metastases. PET/CT images show the diffusely increased uptake of ^18^F-FDG in the following areas: (**A**) in the spine; (**B**) in the right humerus; and (**C**) in the left ribs (red arrows indicate the locations of metastatic lesions). Abbreviations: PET, positron emission tomography; CT, computed tomography; ^18^F-FDG, ^18^F-fluorodeoxyglucose radiotracer.

FDG PET/CT is most helpful when standard staging studies are equivocal or suspicious. FDG PET/CT may also help identify the unsuspected regional nodal disease and/or distant metastases when used in addition to standard staging studies. Clinically, FDG PET/CT is used to evaluate systemic metastases comprehensively. Thirteen patients underwent additional molecular breast imaging for cancer staging. Fifteen focal areas showed mild-to-moderate uptake of ^18^F-FDG (Figure 4A), seven and eight of which were identified in the right or left breast, respectively. Axillary lymph nodes in 11 patients (11/13, 84.6%) exhibited FDG uptake (Figure 4B), and seven (7/13, 53.8%) were identified with distant metastases in the liver, lungs, intercostal muscles, and bones (Figure 5). Final pathological examination confirmed that eight patients had positive metastatic lymph nodes. No patients were also diagnosed with positive lymph nodes by pathological analysis, and ^18^F-FDG PET-CT detected negative findings. Moreover, breast mammography, sonography, MRI, and molecular imaging data were all available in nine patients. Ten lesions were detected by mammography, 11 by sonography, 12 by MRI, and 12 by molecular imaging. More importantly, the breast molecular imaging successfully detected an additional three positive metastatic lymph nodes in accordance with the final pathologic examinations.

### Surgical findings

**Figure 6:**
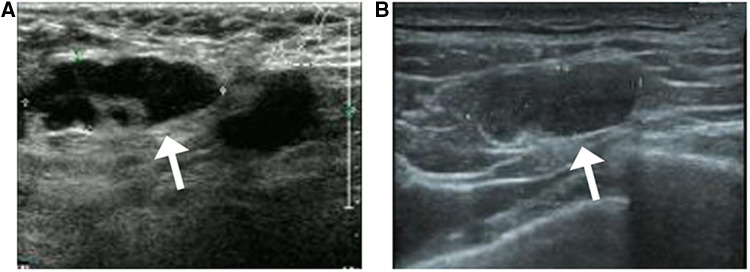
Sonographic findings from a 53-year-old patient with invasive ductal carcinoma with micropapillary features (**A**) axillary sonography showing multiple hypoechoic nodules in the affected axilla, the largest of which is approximately 2.9 cm × 1.1 cm in size, with poorly defined and irregular borders and poorly delineated corticomedullary structures (white arrow) (**B**) multiple hypoechoic nodules are visible in the left supraclavicular fossa, the largest of which measures approximately 1.8 cm × 0.6 cm in size, with poorly defined corticomedullary structures (white arrow).

One patient did not undergo surgical treatment (1/63, 1.5%). Forty-six patients underwent breast-conserving surgery (46/63, 73.1%), and modified radical mastectomy was performed in 16 patients (16/63, 25.3%). Among the 62 patients who underwent surgical procedures, a unifocal lesion was detected in 53 (53/62, 85.5%), whereas multifocal lesions were observed in eight (8/62, 13%), and one had bilateral disease (1/62, 1.6%). Axillary lymph node metastases were detected in 47 of the 62 patients (47/62, 75.8%), eight of whom had micro-metastases. The mean number of metastatic lymph nodes was 19 (range, 10–28). Sonographic analysis revealed a total of 34 patients with abnormal lymph nodes (34/62, 54.8%) (Figure 6), and an ultrasound-guided axillary lymph node fine needle aspiration was performed in 24 patients. Seventeen of these patients had positive lymph node metastases (17/24, 70.8%), five had benign results (5/24, 20.8%) (surgery successfully detected metastatic lymph nodes in three cases), and two had unsatisfactory results (2/24, 8.3%) corresponding to one metastatic lymph node on surgical resection.

### Pathological information

**Figure 7:**
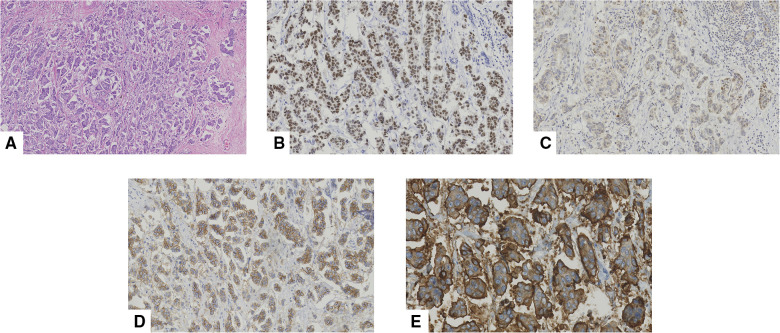
Pathological features of an invasive micropapillary carcinoma (IMPC) in a 43-year-old woman who underwent mastectomy. (**A**) Hematoxylin-eosin (H/E) staining indicated that the breast tumor was composed of morula-like clusters floating in empty, clear spaces lined by delicate strands of stroma (original magnification ×100). Immunohistochemical staining of (**B**) estrogen receptor (ER, original magnification ×200), (**C**) progestogen receptor (PR, original magnification ×200), (**D**) human epidermal growth factor receptor-2 (HER-2, original magnification ×200), and (**E**) Mucin-1 (original magnification ×400), respectively. More than 90% of ER demonstrated strong positive. 10% of PR was diagnosed as weak to moderate intensity positive. HER-2 staining indicated positive (2+). Mucin-1 staining demonstrated “inside-out” pattern was strong positive in cavity margins.

Among the 62 patients who underwent surgery, five had tumors that were characterized as grade 1 (5/62, 8.1%), 43 had grade 2 tumors (43/62, 69.3%), and 14 had grade 3 tumors (14/62, 22.6%). Pathologic specimens were available for all patients. The final pathologic reports demonstrated that the mean size of all lesions was 2.4 cm (range, 0.7 cm–6.1 cm). All imaging and surgical findings of tumor sizes are summarized in Table 5. A ductal carcinoma *in situ* component was found in 47 patients (47/62, 75.8%), and 44 exhibited associated angiolymphatic invasion (44/62, 70.9%). Although nine patients had no data on receptor statuses, positive ER expression was observed in 48 patients (48/53, 90.5%), and positive PR expressions were found in 38 (38/53, 71.6%); however, HER2 positivity was present in only 14 (14/53, 26.4%) (Figure 7).

**Table 5:** Tumor size determined by mammography, sonography, MRI, and surgical pathology in patients with IMPC (*n* = 63).

Patient number	Mammography	Sonography	MRI	Surgical pathology
1	NM	1.3	1.5	1.7
2	NM	3.7	5.8	6.0
3	2.8	2.7	2.5	3.0
4	N	1.8	1.6	1.5
5	2.9	3.4	ND	4.0
6	1.2	1.0	1.6	1.0
7	ND	1.9	1.9	2.0
8	ND	3.7	2.7	4.5
9	1.7	1.8	3.1	2.0
10	ND	2.0	ND	2.0
11	ND	1.0	0.9	1.0
12	ND	2.4	ND	2.0
13	ND	2.8	2.7	3.0
14	ND	2.4	ND	2.5
15	ND	1.9	1.5	2.5
16	ND	1.8	ND	2.0
17	ND	0.8	0.8	1.0
18	ND	1.2	1.5	1.8
19	ND	4.4	ND	3.8
20	ND	1.7	1.5	3.5
21	ND	2.3	2.6	2.7
22	ND	1.9	1.6	2.0
23	ND	3.4	5.9	5.0
24	1.5	1.4	1.4	1.5
25	ND	ND	2.5	2.0
26	ND	1.1	1.3	1.3
27	2.3	2.0	1.6	ND
28	2.2	1.9	2.0	2.0
29	2.0	ND	ND	2.3
30	1.7	2.0	2.2	3.5
31	ND	ND	2.1	2.0
32	3.9	3.6	ND	5.5
33	5.0	3.2	2.8	3.5
34	1.8	2.4	2.7	2.6
35	1.4	1.2	1.3	2.0
36	1.4	1.4	1.2	1.3
37	1.6	ND	1.9	1.5
38	3.2	3.8	ND	5.0
39	1.5	1.7	1.6	1.5
40	3.2	ND	3.0	2.0
41	1.3	1.0	1.2	1.2
42	ND	ND	3.4	3.0
43	3.0	2.6	2.5	2.7
44	1.1	1.7	1.2	1.4
45	6.5	5.0	5.1	6.1
46	ND	1.2	1.6	2.0
47	ND	2.4	2.1	2.2
48	0.9	0.8	0.8	0.7
49	2.1	2.2	1.9	2.4
50	2.5	2.4	ND	2.8
51	2.1	ND	1.8	2.0
52	1.9	2.1	2.0	2.2
53	2.5	2.2	ND	2.4
54	2.0	ND	1.6	1.7
55	1.9	ND	1.5	1.6
56	1.7	1.9	1.7	1.7
57	ND	1.4	1.1	1.2
58	ND	1.7	1.5	1.5
59	ND	2.0	1.8	2.0
60	ND	1.1	1.0	1.3
61	ND	1.2	1.3	1.5
62	ND	1.9	1.4	1.6
63	ND	1.1	1.3	1.6

Notes: Data are tumor size in centimeters.

Abbreviations: MRI, magnetic resonance imaging; IMPC, invasive micropapillary breast carcinoma; N, negative; ND, not done; NM, not measurable.

### Prognostic findings

Forty-one patients had clinical follow-up data, 26 of whom (26/41, 63.4%) did not experience any recurrence during the median follow-up period of 4.3 years (range, 1–14 years). Local regional disease recurrence was found in four patients (4/41, 9.8%), all of whom underwent breast-conserving surgery. One patient experienced two local recurrences in the right breast (1 and 4 years after diagnosis). Nine (9/41, 21%) patients developed distant metastases in the adrenal glands, lung, bone, brain, and mediastinum. Nine patients died after the initial breast cancer diagnosis, whereas four died at 2, 4, 7, and 11 years of causes unrelated to breast disease.

## Discussion

IMPC is a rare and morphologically distinctive subtype of breast carcinoma characterized by the presence of small, hollow, or morula-like clusters of cancer cells, which are surrounded by clear stromal spaces and display a reverse polarity. IMPC exhibits more aggressive clinical behaviors than invasive ductal carcinoma, with more possibilities for local recurrence, lymph node metastasis, regional LVI, and distant metastasis (10, 11). Although IMPC was listed as a subtype of invasive breast carcinoma in 1993, no consensus has been successfully reached on which criteria to use for diagnosis and determining the type of disease based on the percentage of the IMPC component (12). Regardless of the tumor size and the proportion of the IMPC component, a few studies have emphasized the importance of the histologic features in determining the invasiveness of IMPC (4, 13). In our study, data from 63 patients with a micropapillary component ratio ranging from 10% to 100% were reviewed, and the clinical imaging features were highly indicative of the malignancy of IMPC.

The incidence of IMPC in our study was 1.5% among all primary breast carcinomas, which is higher than that reported in a study by Hashmi et al. of 0.67%; (14). However, this value is obviously lower than the incidences of 3% and 7.6% reported elsewhere (11, 15). The mean age of our patients was 56.3 years (ranging from 28 to 90 years), which is younger than that of a previous study performed by Acs et al., which reported a mean age of 59.5 years (16). The mean tumor size of our patients was 2.4 cm (range, 0.7 cm–6.1 cm), whereas Al-Sharif reported a mean size of 2.3 cm (range, 0.3 cm–15 cm), and Cruz et al. reported a size of 3.4 cm (range, 0.8 cm–8.4 cm) (8, 17). No specific side predominance was observed in our study. In addition to two patients diagnosed with bilateral lesions, 20 were on the left, and 23 were on the right. These findings contradict the results reported by Kim et al., who demonstrated a left-side predominance in over 50% of patients (18). The most common clinical manifestation of IMPC is a palpable mass (19); this characteristic is consistent with our findings (30 of 63 patients; 48%). Twenty-six patients suffered from skin changes and nipple retraction, and fourteen (14/26, 53.8%) received neoadjuvant therapy. Before their first neoadjuvant therapy, all patients underwent MRI examinations, which detected suspected abnormal lymph nodes in six patients (6/14, 42.9%), while six (6/14, 42.9%) had benign results, two (2/14, 14.2%) had suspicious results. The final pathologic reports positive lymph node metastases in seven patients (7/14, 50%); two (2/14, 14.2%) were identified with abnormal lymph nodes by MRI examinations before neoadjuvant therapy. However, their final pathologic reports presented a negative result. Given that false-positive findings on breast MRI are common, these two patients were insufficient to demonstrate that neoadjuvant therapy effectively treated lymph node invasion.

As previous studies have reported, the mammographic findings of IMPC strongly indicate this disease's invasiveness. In our study, the most common presentation on breast mammography was an irregular mass. The breast mammograms successfully detected 37 masses with an irregular shape (24/37, 64.8%), a spiculated margin (19/37, 51.3%), and a high density (22/37, 59.4%). These findings are consistent with the results reported by Jones et al., which indicated that the most common morphologic characteristic of IMPC was an irregular mass (50% of cases), frequently with a high density and spiculated margins (42% of cases) (6); however, contradictory results were reported by Günhan-Bilgen et al. that indicated a round or ovoid mass was present in 53.8% of all cases (20). Associated calcifications were present in 12 lesions (10/12, 83.3%), the majority of which displayed a punctate morphology (10/12,83.3%) and a scattered distribution (9/12, 75%). These findings are similar to those reported in studies by Yun and Adrada that showed a fine pleomorphic appearance (66.7% and 68%, respectively) (7, 19).

Meanwhile, the most common sonographic findings of IMPC were hypoechoic masses (50/69, 72.4%) with irregular shapes (61/69, 88.4%), complex posterior acoustic patterns (16/69, 23.1%), and spiculated (38/69, 55.1%) or angular margins (30/69, 43.4%). These sonographic characteristics were consistent with the results reported by Alsharif and Jones et al., both of which indicated that the most frequent features of IMPC were irregularly shaped, hypoechoic, and spiculated masses (6, 8). In our study, no mass displayed an isoechoic pattern, a finding that strongly opposed those reported by Kamitani et al. showing that 50% of masses were isoechoic (21). The subsequent color Doppler interrogation detected 30 lesions showing punctate blood flow (30/52, 57.6%), which was lower than the percentage reported by Alsharif et al. of 68.7% of masses with increased vascularity (8). For the axillary sonographic examination, 54 patients underwent axillary sonography, 34 of whom exhibited abnormal lymph nodes (34/54, 62.9%). An ultrasound-guided axillary lymph node fine needle aspiration was performed in 24 patients, 17 of whom had positive lymph node metastases (17/24, 70.8%), five had benign results (5/24, 20.8%) (surgery successfully detected metastatic lymph nodes in three patients), and two experienced unsatisfactory results (2/24, 8.3%) corresponding to one metastatic lymph node on surgical resection; these rates were slightly higher than the abnormal axillary ultrasound rate reported by Günhan-Bilgen et al. (38% of patients) (20) but were similar to that reported by Adrada et al. (48% of patients) (8). Only Jones et al. showed that 67% of patients had sonographic findings indicative of axillary lymphadenopathy (6).

In recent years, several studies have concentrated on the MRI features of IMPC. These studies all demonstrated that the most common presentation of IMPCs was an irregular, spiculated mass with early rapid initial heterogenous enhancement in breast MRI (7, 22), indicating that the MRI findings correlated with the invasiveness of IMPC. In our study, the most common findings were irregularly shaped masses (53/68, 77.9%), with irregular or spiculated margins (23/68, 33.8%; 30/68, 44.1%), and type II or III kinetic curves (37/68, 55%; 27/68, 40%). These findings are similar to the results from those previous studies. Furthermore, a total of 52 patients were matched in our study, and MRI identified a total of 68 lesions; the number of cases was higher than those included in the series by Jones and by Alsharif et al., which had 18 and 13 patients, respectively (6, 8). In addition, we noted that 42.5% of the masses displayed heterogeneous enhancement in breast MRI. This finding was contrary to that of the previous report by Alsharif et al., which demonstrated that most masses displayed homogeneous enhancement (8). No non-mass-like enhancement was observed, whereas Yun and Jones et al. reported seeing it (6, 7).

Currently, there are only two previous studies concerning breast molecular imaging findings of IMPC (6, 7). Only one patient was included in a clinical study performed by Jones et al., which identified mild-to-moderate radiotracer uptake in two focal areas in the right breast. Meanwhile, Yun et al. analyzed the uptake of ^18^F-FDG in 16 patients, and PET/CT detected ^18^F-FDG uptake in all primary breast cancer lesions. In our study, 13 patients underwent ^18^F-FDG for clinical cancer staging. We also found 15 focal areas that exhibited a mild-to-moderate FDG uptake, consistent with the findings of Yun et al.

Previous studies reported that IMPC is an intensely aggressive subtype of breast cancer with a high possibility of spreading to the lymph nodes and through the lymphatic system. Angiolymphatic invasion is considered an important prognostic factor for adverse outcomes and is associated with lymph node metastasis in IMPC (23). In our study, we found that 70.9% of cases exhibited angiolymphatic invasion; our finding is concordant with the research performed by Jones et al., in which angiolymphatic invasion was observed in 69% of patients (6). Meanwhile, breast sonographic examinations detected 54.8% of cases with suspected abnormal lymph nodes; this rate was higher than that reported by Günhan-Bilgen et al. but lower than that of Jones et al., which were 38% and 67%, respectively (6, 20). In addition, our study showed that 70.8% of patients were diagnosed with true-positive metastatic lymph nodes, which was comparable to the rate of 69% reported in a previous study by Adrada et al. (19).

Most IMPCs are characterized by strong ER and PR positivity, associated with better tumor differentiation and prognosis (24). This study showed a high incidence of ER (48/53, 90.5%) and PR (38/53, 71.6%) positivity. Our findings are comparable to those reported by Walsh et al., who found ER and PR positivity rates of 90% and 70%, respectively (24). However, we observed a relatively lower incidence of HER-2 overexpression (26.4%) compared with that of a recent study performed by Perron et al., which demonstrated that 65% of IMPCs were HER-2 positive. (25) In addition. In this study, 26 patients (26/41, 63.4%) did not experience any recurrence during the median follow-up period of 4.3 years. This result is obviously higher than the findings of Pettinato et al., who reported a rate of recurrence of 71% (26), but it is lower than that of a study by Jones et al. in which 75% of patients had no recurrence (6). The mortality rate in our study was 12.1%, which was lower than the 37% reported by More et al. (27).

Some limitations were noted in our study that should be acknowledged. Firstly, the retrospective nature of the study meant that it could not sufficiently identify all of the imaging and pathologic characteristics of IMPC due to the fact that not all data was available for each patient. Secondly, we directly reviewed our institution's surgical pathology database and identified the clinical findings of IMPC while ignoring the impact of the different ratios of micropapillary components in each case and the lack of an additional contrast group composed of patients with other breast cancer subtypes; considering those factors would help facilitate improved precision of imaging and the identification of pathological findings of IMPC. Finally, the rarity of IMPC limited the number of patients that could be included in this study. Multicenter studies with larger sample sizes should be conducted to allow for further comprehensive evaluation of the imaging and pathological characteristics of IMPC.

## Conclusion

Imaging characteristics of IMPC based on mammography, sonography, MRI, and breast molecular imaging were highly indicative of malignancy. The presence of invasive ductal carcinoma with different proportions of micropapillary components is intensely associated with LVI, lymph node metastases, and a high possibility of ER and PR positivity. These findings are important for helping radiologists and pathologists distinguish IMPC from other breast diseases and may contribute to the comprehensive treatment and prognostic prediction of IMPC.

## Data Availability

The raw data supporting the conclusions of this article will be made available by the authors, without undue reservation.
